# Accuracy of computer-aided ultrasound as compared with magnetic resonance imaging in the evaluation of nonalcoholic fatty liver disease in obese and eutrophic adolescents*

**DOI:** 10.1590/0100-3984.2014.0074

**Published:** 2015

**Authors:** José Hermes Ribas do Nascimento, Ricardo Bernardi Soder, Matias Epifanio, Matteo Baldisserotto

**Affiliations:** 1 PhD, MD, Radiologist and Teacher at Instituto Cenecista de Ensino Superior de Santo Ângelo (IESA), Santo Ângelo, RS, Brazil.; 2 PhD, Neuroradiologist at Instituto do Cérebro – Pontifícia Universidade Católica do Rio Grande do Sul (InsCer-PUCRS), Porto Alegre, RS, Brazil.; 3 PhD, Coordinator for Pediatrics Internship, Pontifícia Universidade Católica do Rio Grande do Sul (PUCRS), Porto Alegre, RS, Brazil.; 4 PhD, Coordinator for the Imaging Center of Instituto do Cérebro – Pontifícia Universidade Católica do Rio Grande do Sul (InsCer-PUCRS), Porto Alegre, RS, Brazil.

**Keywords:** Steatosis, Ultrasound, Obesity, Adolescents

## Abstract

**Objective:**

To compare the accuracy of computer-aided ultrasound (US) and magnetic resonance imaging (MRI) by means of hepatorenal gradient analysis in the evaluation of nonalcoholic fatty liver disease (NAFLD) in adolescents.

**Materials and Methods:**

This prospective, cross-sectional study evaluated 50 adolescents (aged 11–17 years), including 24 obese and 26 eutrophic individuals. All adolescents underwent computer-aided US, MRI, laboratory tests, and anthropometric evaluation. Sensitivity, specificity, positive and negative predictive values and accuracy were evaluated for both imaging methods, with subsequent generation of the receiver operating characteristic (ROC) curve and calculation of the area under the ROC curve to determine the most appropriate cutoff point for the hepatorenal gradient in order to predict the degree of steatosis, utilizing MRI results as the gold-standard.

**Results:**

The obese group included 29.2% girls and 70.8% boys, and the eutrophic group, 69.2% girls and 30.8% boys. The prevalence of NAFLD corresponded to 19.2% for the eutrophic group and 83% for the obese group. The ROC curve generated for the hepatorenal gradient with a cutoff point of 13 presented 100% sensitivity and 100% specificity. As the same cutoff point was considered for the eutrophic group, false-positive results were observed in 9.5% of cases (90.5% specificity) and false-negative results in 0% (100% sensitivity).

**Conclusion:**

Computer-aided US with hepatorenal gradient calculation is a simple and noninvasive technique for semiquantitative evaluation of hepatic echogenicity and could be useful in the follow-up of adolescents with NAFLD, population screening for this disease as well as for clinical studies.

## Abstract (pt)

**Objetivo:**

Comparar a acurácia do ultrassom (US) com análise computadorizada mediante cálculo do gradiente hepatorrenal na avaliação da doença hepática gordurosa não alcoólica (DHGNA), utilizando a ressonância magnética (RM) como referência.

**Materiais e Métodos:**

Este estudo, transversal prospectivo, avaliou 50 adolescentes (idades de 11 a 17 anos), sendo 24 deles obesos e 26 eutróficos. Todos os adolescentes foram submetidos a exames de US com análise computadorizada, RM, exames laboratoriais e avaliação antropométrica. Foram calculados sensibilidade, especificidade, valores preditivos positivos e negativos e acurácia, seguindo a posterior geração de curva ROC (receiver operating characteristic) e cálculo da área sob a curva estabelecida, para determinar o melhor ponto de corte para o gradiente hepatorrenal, para predizer graus de esteatose, utilizando os resultados da RM como padrão ouro.

**Resultados:**

Resultados: No grupo de obesos, 29,2% foram meninas e 70,8% foram meninos, e no grupo de eutróficos, 69,2% foram meninas e 30,8% foram meninos. A prevalência de DHGNA foi 19,2% nos eutróficos e 83% nos obesos. A curva ROC gerada para o gradiente hepatorrenal, com ponto de corte de 13, apresentou sensibilidade de 100% e especificidade de 100%. Considerando o mesmo ponto de corte para os eutróficos, haveria 9,5% de falso-positivos (especificidade de 90,5%) e 0% de falso-negativos (sensibilidade de 100%).

**Conclusão:**

O US com análise computadorizada, com o cálculo do gradiente hepatorrenal, é técnica simples e não invasiva para avaliação semiquantitativa da ecogenicidade hepática e poderá vir a ser de ajuda no seguimento de adolescentes com DHGNA, no rastreamento populacional dessa doença e para estudos clínicos.

## INTRODUCTION

Several factors have stimulated the development and improvement of researches about noninvasive methods for the diagnosis of nonalcoholic fatty liver disease (NAFLD), a theme of great interest in medicine^(1)^.

Over the last decades, due to the increase in the rates of prevalence of overweight and obesity, the remarkable increase of NAFLD as the main cause of hepatic disease in the pediatric population worldwide is justified^(2-4)^. In the United States of America, a drastic increase has been observed in the number of cases of NAFLD possibly progressing to more severe conditions such as steatohepatitis, cirrhosis and hepatocarcinoma, with estimated 80 to 100 million Americans affected^(5-9)^. In Europe, the number of cases has increased between 10% and 40% over the past 10 years^(10)^. Studies on childhood obesity utilizing clinical, biochemical and sonographic parameters for grading fatty infiltration of the liver recorded hepatic steatosis prevalence between 53% and 77%^(11,12)^. It is a known fact that the prevalence and severity of NAFLD are related to ethnic factors, increasing in the Mexican American children population as well as in Hispanicorigin populations as compared with other ethnic groups^(13)^.

Hepatic steatosis is a broad term corresponding to accumulation of lipids, especially triglycerides, in the hepatocytes cytoplasm, exceeding 5% of the liver weight^(14,15)^. Nonalcoholic fatty liver disease comprises a wide spectrum of variations where steatosis plays a fundamental role in the development of the disease, progressing to fibrosis in up to 41% of the cases according to several studies^(7,16,17)^.

Currently, there is no specific biochemical marker or serological testing for the diagnosis of NAFLD, and liver biopsy is the most accurate diagnostic method providing important data on the degree of hepatic compromising, global changes in the liver architecture and evaluating the severity of the inflammatory process and fibrosis^(18)^. However, due to the invasive nature of liver biopsy, it cannot be extensively utilized for large scale population screening purposes, not even in the post-treatment follow-up of patients due to the risks associated with the procedure^(19)^.

Up to this moment, different imaging techniques have been utilized to detect hepatic steatosis, as follows: ultrasonography (US), computed tomography (CT), magnetic resonance imaging (MRI) and magnetic resonance spectroscopy (MRS)^(19,20)^. CT has a good accuracy, with semiquantitative diagnosis, but its utilization in the monitoring of treatment response is limited because of the utilization of ionizing radiation^(21)^. MRI is currently the most accurate technique, capable of detecting fat amounts below 0.5%, but it presents some limitations – the extended time for the clinical routine, high cost, required operator experience, data processing and results interpretation – limiting its utilization in the clinical practice for population studies and treatment monitoring^(21,22)^.

US is a low-cost method, but it relies on a subjective analysis for the evaluation of steatosis, i.e., it does not provide quantitative data, thus its sensitivity is reduced by up to 60%, particularly in obese patients or in those with little fatty infiltration in the liver^(12,23)^.

Recently, Soder et al. described a new technique for the diagnosis of NAFLD relying on US utilizing semiquantitative computed analysis, with the calculation of the hepatorenal gradient, which presented with excellent results in obese and eutrophic children^(24)^. However such a technique was not compared with any other imaging method.

It is important to highlight that up this moment, no clinical studies have been undertaken comparing the accuracy of computer-aided US hepatorenal gradient with MRI that is the gold standard for images analysis in the diagnosis of NAFLD.

As computer-aided US is a reproducible and low-cost method in the evaluation of NAFLD, the present study was aimed at comparing the accuracy of computer-aided US with hepatorenal gradient calculation with the quantification obtained by means of MRI in the investigation of NAFLD in obese and eutrophic adolescents, with the objective of validating the technique.

## MATERIALS AND METHODS

### Study population

Prospective, cross-sectional study developed in the period from October, 2011 to February, 2012 involving 50 adolescents (ages from 11 to 17 years). A group of 24 obese adolescents assisted in a nutrition ambulatory service, and another group of eutrophic adolescent students, volunteers from a public school, were invited to participate in the study.

The study was duly approved by the Committee for Ethics in Research of the Institution and terms of free and informed consent were signed by the parents or legal caretakers of the adolescents.

The adolescents presenting with hepatorenal disease, or undergoing treatment with hepatotoxic, nephrotoxic, corticosteroid or immunosuppressant drugs were excluded from the study, as well as those presenting with other chronic diseases.

The adolescents were submitted to US and MRI, laboratory tests and anthropometric evaluation.

The selected patients were examined by a nutritionist with a ten-year experience, bearing a specialist title. The physician coordinator of the multidisciplinary team requested the laboratory tests, which were obtained up to one week after the anthropometric measurements, US, MRI scans.

The computer-aided US scans were performed by a radiologist with an extensive experience in the field of abdominal radiology, bearing an American-equivalent specialist title, utilizing a Voluson (GE Healthcare; Milwaukee, USA) US apparatus. On the same day, the MRI scans were performed in a 1.5 T Signa Excite (GE Healthcare; Milwaukee, USA) apparatus, equipped with phased array surface coils, fourchannel column array coils.

### Anthropometric measurements

Data on gender, age, weight, height and abdominal circumference were collected. Body mass index (BMI) table of the National Center for Health Statistics was utilized in order to access the percentile for each adolescent. The Z score was calculated for the purpose of comparing the BMI at different ages and genders. The Z score represents the number of standard deviations above and below the mean values of the population in the childhood and adolescence.

Body mass index (BMI) ≥ the 97th percentile was utilized for obesity analysis, while for the eutrophic group, the BMI between the 25th and 75th percentiles were utilized. The BMI was calculated by dividing weight (kg) by the squared height (m^2^). In order to compare the BMI at different ages and genders, the Z score > +2 corresponded to diagnosis of obesity.

### Computer-aided US of the liver

Grayscale, real time US scans were performed with a 3.5–5 MHz convex transducer coupled with a GE platform with image parameters adjusted for all examined adolescents. Two images representative of the hepatic parenchyma and of the right renal cortex were obtained from each patient, by utilizing a subcostal approach in the hemiclavicular line. The images comprised the VI hepatic segment and the adjacent renal parenchyma, obtained in the area of the hepatorenal space, during maximal inspiration. After digitalization of the images acquired in the DICOM format, the Image J software was utilized for computer-aided analysis, measuring the echogenicity of the digital US image. This software was utilized to quantify the echogenicity of the hepatic parenchyma and of the right renal cortex, with the values being graded in grayscale ranging from 0 to 255, from black to white, respectively. Two round-shaped regions of interest (ROIs) were selected, as follows: a) hepatic parenchyma, adjacent to its lower border (area: 400–500 pixels), and right renal cortex adjacent to its upper border (area: 300–400 pixels) selected in a juxtaposed manner and under the same axis of the acoustic beam, with the purpose of minimizing artifacts and variations in echogenicity of the acoustic window, a method previously described by Soder et al.^(24,25)^.

**Figure 1:**
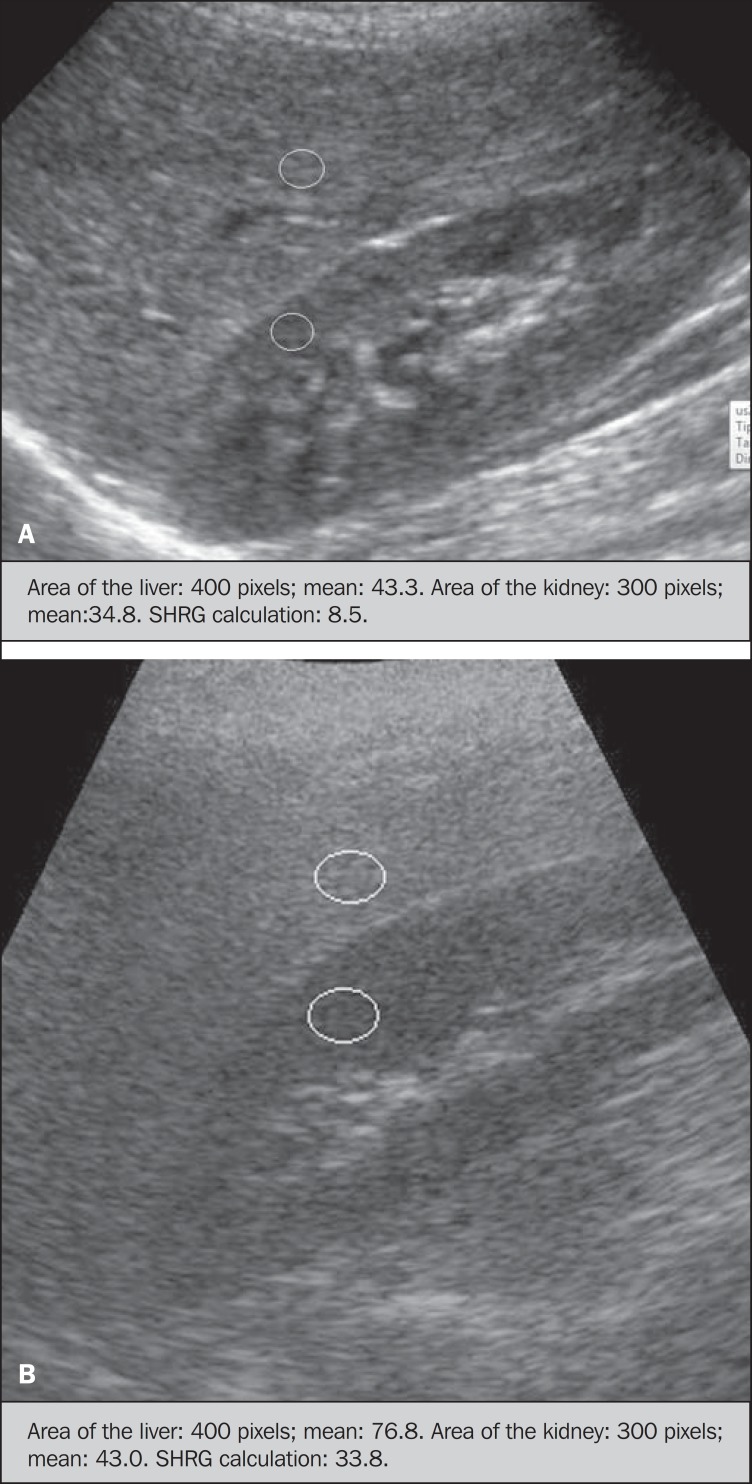
Sonographic images demonstrating the regions of interest. **A:** Liver US of an eutrophic adolescent. Hepatorenal gradient (without steatosis). **B:** Liver US of an obese adolescent. Hepatorenal gradient (with steatosis).

Once the echogenicity in the ROIs was measured, the two resulting values were subtracted in order to obtain the sonographic hepatorenal gradient (SHRG). Such a protocol was applied to all adolescents included in the present study, either obese or eutrophic (Figure 1).

The DICOM images were recorded and were analyzed by two radiologists in order to check the consistency of the values.

### Liver MRI technique

Liver images of the eutrophic and obese patients were obtained in a MRI 1.5 T apparatus with a GE platform. The images were acquired in the axial plane at the end of expiration, under breathhold, in order to globally reduce the acquisition time to approximately 15 s. For the calculation of the fat fraction (FF) the Dixon two-point method, modified by Fishbein et al.^(23)^ was utilized, as described in the study by Pacifico et al.^(25)^. Such a method is based on the imaging phase change, where the FF is calculated by the signal difference between two vectors resulting from the in-phase and out-of-phase signal (Table 1).

**Table 1:** MRI accuracy in the diagnosis of mild, moderate and severe steatosis, according to Pacifico et al.(25).

		Hepatic steatosis	
	≥ 5%	≥ 33%	≥ 66%
	Grade I - mild	Grade II - moderate	Grade III - severe
Cutoff point	4.85	9	19
Area over the curve	0.98*	1	1
Sensitivity	95.8%	100%	100%
Specificity	100%	100%	100%

* CI 95%: 0.98-1.0.

The parameters for multiple T1-weighted gradient echo sequences were the following: repetition time, 174 ms; echo time, 2.1 ms for out-of-phase images and 4.9 ms for in-phase signals; field of view, 35 cm × 40 cm; slice thickness, 5 mm; 70° flip angle; matrix size, 256 × 182. Pixels for in-phase and out-of-phase signal intensity were obtained in order to select the ROIs. Signal intensity values for the liver and spleen were acquired in-phase and out-of-phase, with a mean value of the circular ROIs area corresponding to 1 cm^2^^(25,26)^.

The hepatic fat was quantified as a relative percentage of the liver signal intensity loss at the out-of-phase images, by means of the following equation: 

FF=(SIip−SIop/2×SIip)×100

where: SI is the mean value of the liver signal intensity divided by the mean value of the spleen signal intensity; SI ip and SI op are the in-phase and out-of-phase signal intensities respectively. The spleen signal intensity was utilized as a denominator in the equation to adjust the lack of an objective MRI signal intensity scale, method utilized by Pacifico et al.^(25-27)^. 

**Figure 2:**
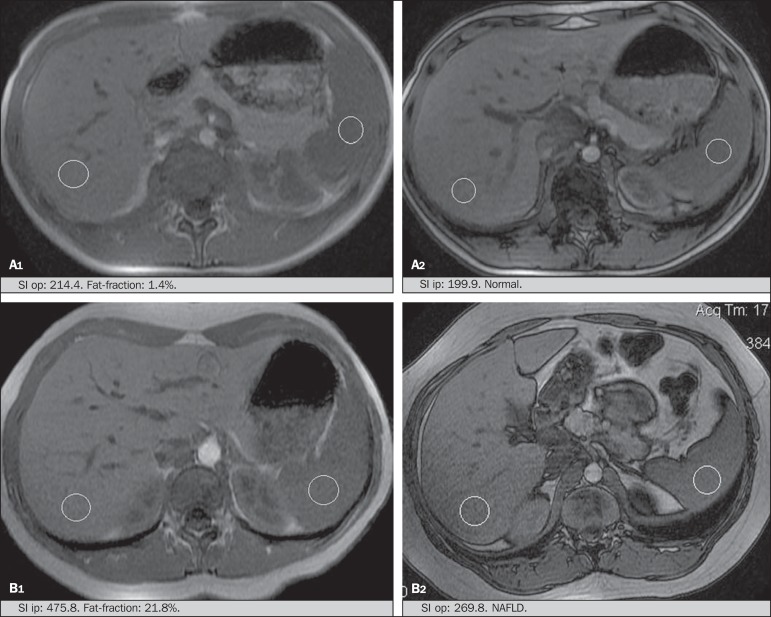
**A:** MRI in-phase , out-of-phase and chemical shift images demonstrating signal intensity and regions of interest in a patient without steatosis. **B:** MRI inphase, out-of-phase and chemical shift images of a patient with steatosis demonstrating signal intensity and regions of interest.

A drawing of the ROIs was made on the VI liver segment, adjacent to the renal cortex, corresponding to the same segment scanned at US, carefully avoiding vessel areas, motion artifacts and partial volume effect. Similar measurements of the spleen were made and the average signal intensity was calculated for the liver ROIs (Figure 2).

### Statistical analysis

The data were recorded, demonstrated on charts and tables and were then analyzed by statistical methods, and recorded on Excel worksheets. The SPSS (Statistical Package for the Social Sciences) software release 18.0 was utilized.

Continuous data were presented in mean and standard deviations (symmetrical distribution case) or median and interquartile range (asymmetrical distribution case). Categorical data were presented as absolute frequencies and proportions.

The Friedman’s test supplemented by the intraclass correlation coefficient was utilized to evaluate the interobserver agreement; and the intraobserver agreement was evaluated by means of the Wilcoxon’s test, also supplemented by the intraclass correlation coefficient.

Differences between mean values were evaluated by means of the Student’s t test for variables with normal distribution, and by means of the Mann-Whitney’s test in the case of abnormal distribution. The chi-squared test was utilized to evaluate the categorical variables.

In order to evaluate the sonographic gradient diagnostic properties, sensitivity, specificity, positive and negative predictive values and accuracy were calculated, followed by the generation of the receiver operating characteristic curve (ROC) and calculation of the area under the curve, in order to determine the best cut-off point for the hepatorenal gradient, for the prediction of steatosis degree utilizing the MRI results as gold standard. The selected cutoff points were focused on the minimization of false-positive and false-negative values.

The *p* < 0.05 value was considered as being statistically significant.

### Calculation of sample size

For the purpose of sample size calculation, data from previous studies evaluating accuracy and quantifying fat in the childhood by means of MRI^(13,24,28)^ were utilized. Based on such studies, by adopting FF estimated by MRI and sonographic gradient for a linear correlation coefficient (Pearson’s r) in the order of 0.5, significance level of 5% and 90% statistical strength, 50 individuals are necessary^(5,24)^. 

## RESULTS

The obese group included 7 girls (29.2%) and 17 boys (70.8%), while the eutrophic group included 18 girls (69.2%) and 8 boys (30.8%). The mean age in the obese group was 14.2 years (± 2; 11–17 years), and in the eutrophic group it was 14.7 years (± 2; 12–17 years). In the obese group there was a significantly higher proportion of boys as compared with the eutrophic group (*p* = 0.011).

The proportion of cases of increased abdominal circumference was statistically significant between the eutrophic and obese groups, respectively 15.4% versus 95.8%; *p* < 0.001).

In the present study, steatosis was found in 5 (19.2%) eutrophic patients, while in the obese group 20 individuals (83%) presented with steatosis.

The prevalence of steatosis in the obese group was significantly higher (83.3%; CI 95%: 64.5–94.5) than in the eutrophic group (19.2%; CI 95%: 7.4–37.6).

**Figure 3:**
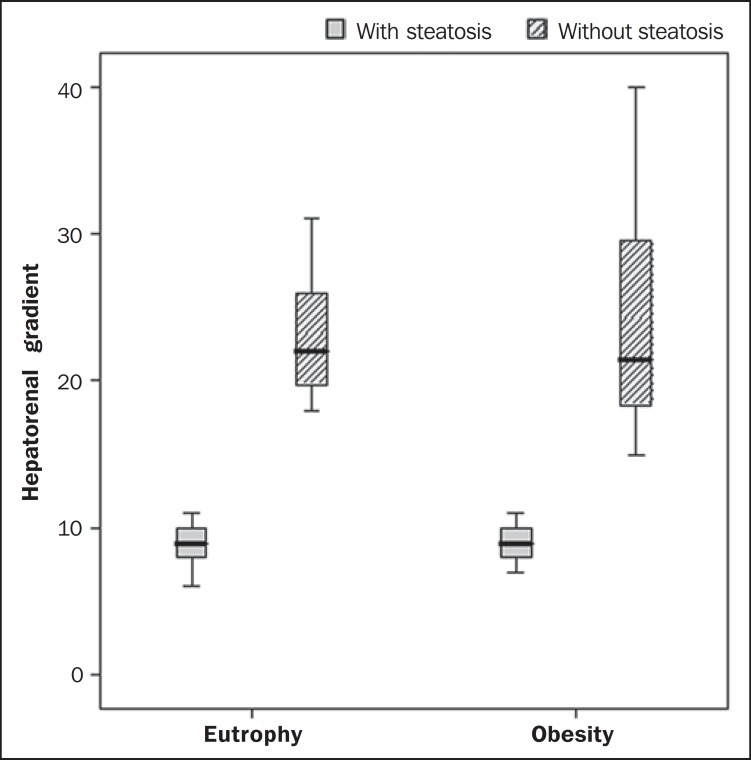
Box plot depicting gradient values (levels) between groups of individuals with and without steatosis. The central line within the box represents the median. The upper and lower limits of the box represent respectively the 75th and 25th percentiles. The upper and lower error bars represent the median ± 1.5 (P75–P25).

After SHRG analysis, whose calculation was based on the echogenicity difference between the liver cortex and the renal parenchyma, a significant difference was observed between the median of the obese adolescents and of the eutrophic ones (median = 19.5; P25 = 15.5; P75 = 28 versus median: 10.0; P25 = 8; P75 = 11; *p* < 0.001). However, as the adolescents presenting with steatosis were separated from those without steatosis, and the difference between obese and eutrophic adolescents was reevaluated, no statistically significant difference was observed (with steatosis: *p* = 0.818; without steatosis: *p* = 0.971) (Figure 3).

Also, a statistically significant difference was observed between the medians of adolescents with and without steatosis (median = 22; P25 = 18.5; P75 = 29.5 versus median = 9; P25 = 7.8; P75 = 10; *p* < 0.001).

**Figure 4:**
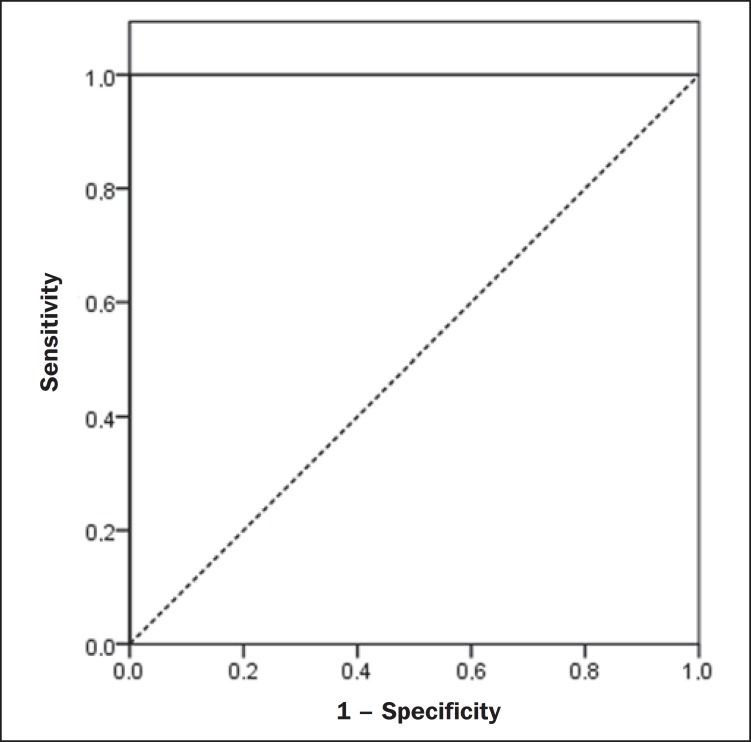
ROC curve in the evaluation of the cutoff point for the SHRG, considering MRI as the gold standard in steatosis.

The ROC curve generated for the hepatorenal gradient is demonstrated on Figure 4, with a cutoff point of 13, with 100% sensitivity and 100% specificity (Table 2). Considering this same cutoff point for the eutrophic adolescents, there would be 9.5% of false-positive cases (90.5% specificity) and 0% of false-negative cases (100% sensitivity).

**Table 2:** Computer-aided US accuracy utilizing MRI as gold standard.

Hepatic steatosis	Total sample	Eutrophic adolescets	Obese adolescents
Cutoff point	13.0	14.5	13.0
Area over the curve	1.00 (1.00-1.00)	1.00 (1.00-1.00)	1.00 (1.00-1.00)
Sensitivity	100%	100%	100%
Specificity	100%	100%	100%
Accuracy	100%	100%	100%

After computer-aided analysis of the digital images, no statistically significant difference was found between the echogenicity values of the hepatic parenchyma for the obese adolescents and for the eutrophic ones (87.6 ± 25.5 versus 82.5 ± 32; *p* = 0.535), as well as no statistically significant difference was observed in the renal echogenicity of both groups (67.3 ± 31.2 versus 73.9 ± 32.5; *p* = 0.466).

### Validation of the computer-aided US technique

The US scans performed in 50 patients included in the study were randomly separated and reevaluated by three radiologists, all of them with more than 10 years of experience in abdominal radiology, for the calculation of SHRG. The US scans results validated the SHRG, demonstrating that there was no significant difference between the three radiologists (Friedman’s test; *p* = 0.100), and with high intraclass correlation coefficient (ICC = 0.95; IC 95%: 0.90–0.98).

Also, no difference was observed either between the two evaluations by the same observer (Wilcoxon’s test; *p* = 0.275), and with high intraclass correlation coefficient (ICC = 0.97; IC 95%: 0.93–0.99).

## DISCUSSION

The imaging evaluation of the liver and of the biliary tract, particularly by US, has been the subject of a series of recent publications in the Brazilian radiological literature^(29-34)^.

In the present study, it was demonstrated that the prevalence of NAFLD was 19.2% in the eutrophic group and 83% in the obese group, similarly to the study developed by Chan et al., who have found a prevalence of hepatic steatosis between 53% and 77%^(12)^. However, in the study developed by Pacifico et al. with 55 obese Italian children, a steatosis prevalence of 40% was found, diagnosed by means of MRI^(28)^. According to Devadason et al., in a comprehensive review approaching NAFLD and non-alcoholic steatohepatitis, limited studies on the prevalence of such diseases in children and adolescents were found on account of the practical difficulties in diagnosing steatosis, which is defined by means of invasive biopsy and costly imaging methods such as MRI, that are not routinely performed in the clinical practice^(35)^.

The study developed by Soder et al. has evaluated a new method in the attempt to quantify NAFLD – computer-aided US utilizing a software (Image J), which can quantify the digital images obtained by the US apparatuses and therefore, the echogenicity of the hepatic and renal parenchyma could be measured for the gradient calculation. In such study, both obese and eutrophic children were evaluated, with significant differences in SHRG medians between the two analyzed groups; however, such a diagnostic method was not compared with any other imaging method^(24)^.

In fact, in the present study, the SHRG calculation revealed a significant difference between the medians of the obese adolescents and the eutrophic ones (19.3 versus 10.0; *p* < 0.001), similarly to the study developed by Soder et al. (33.9 ± 6.6 versus 14.1 ± 6). A significant difference was observed between the medians of adolescents with and without steatosis (22 versus 9; *p* < 0.001). However, as the adolescents were divided into groups with and without steatosis, and the difference between the obese and the eutrophic adolescents was reevaluated, no significant difference was identified between the two groups (with steatosis: *p* = 0.818; without steatosis: *p* = 0.971). The calculation of the ROC curve generated for SHRG defined a cutoff point of 13, with 100% sensitivity for the prediction of steatosis. Considering this same cutoff point for the eutrophic adolescents, there would be 9.5% of false-positive cases (90.5% specificity) and 0% of false-negative cases (100% sensitivity). The accuracy of the method was 100%. The present study was similar to the one developed by Marshall et al., that defined the cutoff point > 12.7% for steatosis, by utilizing the Image J software with 100% sensitivity. Marshall et al. have validated the hepatorenal index for US in NAFLD, as compared with liver biopsy^(36)^.

Webb et al. have developed a study on patients presenting with chronic liver disease based on liver biopsies, obtaining a cutoff point for the prediction of steatosis > 5% of 14.9, with 100% sensitivity and 91% specificity; the cutoff point to predict steatosis ≥ 25% was 18.6, with 90% sensitivity and 90% specificity. The optimum cutoff point for the hepatorenal index in the prediction of steatosis ≥ than 60% was 22.3, with 90% sensitivity and 93% specificity, also similar to that in the present study^(37)^.

In a study developed by Saadeh et al. utilizing conventional US, subjective, high sensitivity for detecting severe steatosis (> 33%) was demonstrated, as well as 62% positive predictive value and a poor interobserver agreement for mild to moderate steatosis^(38)^. On the other hand, in the study developed by Strauss et al., poor interobserver and intraobserver agreement were evidenced (respectively kappa = 0.43 and kappa = 0.54) at conventional US^(39)^.

Fishbein et al. have observed excellent correlation in the quantification of hepatic fat by means of liver biopsy (scoring for steatosis) and MRI, in adults with predominantly macrovesicular steatosis associated with NAFLD^(23)^. In a study developed by Pacifico et al., MRI and US were quite correlated in what regards microscopic fat, but, although severe steatosis was noticeable at US in all cases, the method could not delineate the hepatic fat content. In spite of the fact that US and MRI estimates for steatosis correlate positively, the US limitations in relation to steatosis grading were also observed as compared with the quantitative evaluation of FF by means of MRI^(28)^. Additionally, the fat scoring varied between US and MRI. In children whose US scan revealed moderate to severe steatosis, MRI delineated a wide range of hepatic fat contents within both categories. That means that the usefulness of US seems to be limited by its incapacity to identify either regression or progression of fat in individuals presenting with NAFLD. Thus, if a child presenting with NAFLD undergoes a 40% to 20% decrease in FF at MRI by means of a successful intervention, a corresponding change at US would be improbable^(28)^.

In the present study, because of the small number of patients (25 adolescents) with NAFLD diagnosed by MRI, it was not possible to grade NAFLD as mild, moderate or severe with the calculation of SHRG (sonographic hepatorenal gradient). The authors believe that in further studies with larger patient samples, it will be possible, by means of SHRG calculation, to grade NAFLD as mild, moderate and severe at US, as it has been done at MRI, by means of the FF calculation.

The authors have observed that isolated measurements of the echogenicity of the liver (*p* = 0.534) and of the renal cortex (*p* = 0.466) were not significant among the obese and eutrophic individuals. However, as the SHRG was calculated (difference between echogenicity of the liver and of the renal cortical) a statistically significant difference was observed between the obese and eutrophic groups (19.3 versus 10.0; *p* < 0.001). This can be explained by the fact that there is little or no effect of obesity on the renal cortex, which, differently from the liver, is infiltrated by fat, making the renal cortex a constant parameter for the SHRG calculation.

A limiting factor in the present study was the small number of obese and eutrophic patients in the analysis, hence the non-inclusion of overweight patients in the study. Another problem is the fact that when studying an altered renal parenchyma in association with a pre-existing parenchymal disease, such a disease may affect the US evaluation and the SHRG calculation, as compared with. Similarly, the presence of hepatic fibrosis in some patients, may affect the reliability of the linear correlation between fat infiltration and renal echogenicity.

In the diagnosis of NAFLD, the calculation of the SHRG presented a high accuracy in the group of studied adolescents, suggesting that such a method can reduce the high interobserver and intraobserver variability. A study developed by Jeong et al. with the same proposition, evaluating the relevance of hepatic and renal echogenicity in 54 patients presenting with steatosis has classified the disease as mild, moderate and severe; in patients without steatosis; however, those authors utilized a grayscale histogram with complex parameters and calculations of difficult applicability in the daily clinical practice, but with good results for sensitivity and specificity, as the scores for steatosis were compared^(40)^.

## CONCLUSION

Considering the simplicity and noninvasiveness of computer-aided US with Image J and SHRG calculation for quantitative analysis of hepatic echogenicity, such a method may become helpful in the follow-up of obese and eutrophic adolescents presenting with NAFLD, requiring a short scanning time, without exposing patients to ionizing radiation as in CT, and with reduced costs for the public health system. The method may also become useful in population screening for NAFLD and for clinical studies.
